# ‘*Conceptualizations and implementation of user engagement in Weather and climate services: A climateservicesproviders’ perspective* by Di Fant, V., del Pozo, M., Gulikers, J. and Paparrizos, S

**DOI:** 10.1016/j.heliyon.2023.e22940

**Published:** 2023-12-12

**Authors:** Valeria Di Fant, Maria del Pozo, Judith Gulikers, Spyridon Paparrizos

**Affiliations:** a Deltares (Delft, the Netherlands); b Wageningen University & Research – Environmental Sciences group, Wageningen, the Netherlands; c Wageningen University & Research – Social Sciences group, Wageningen, the Netherlands

**Keywords:** Climate information services, User engagement, Co-production, Co-creation

## Abstract

Weather and Climate Information Services are increasingly used worldwide to facilitate the provision of information to their intended users. Despite this, the definition, classification and evaluation of climate services remains highly debated, particularly regarding user engagement. High forms of user engagement like co-production and co-creation are the least understood. This study looks at three case studies to clarify the current understanding of user engagement in climate services. The research identifies explicit and implicit conceptualizations of user engagement by service providers and characterizes their implementation. The study confirms the current lack of clarity for providers regarding the terminology used to describe user engagement in climate services, which calls for a different understanding of user engagement that allows to better embrace its complexity. Furthermore, our findings reveal that the highest form of user engagement occurred in the case study where there was a shared understanding of how service providers conceptualized user engagement. This conceptualization was aligned with the actual user engagement strategies implemented in the project. This stresses the importance of a shared understanding of user engagement terminology. Finally, the same service is often found to have implemented different user engagement levels at different stages and for different elements of the products. This brings up the issue of how to best describe user engagement in such situations. We recommend embracing the multi-faceted nature of user engagement in climate services by characterizing different elements and stages differently.

## Highlights

• Shared understanding of user engagement terminology is currently lacking.
• Shared terminology among providers can prevent overestimating planned user engagement.
• One service can be characterized by multiple forms of user engagement.
• When characterizing user engagement in CIS, its complexity should be embraced.

## 1 Introduction

Climate Information Services (CIS) provide useable and actionable climate information to users [1]. While their use has been increasing, the definition, classification and evaluation of CIS-related products and activities still remain highly debated [2]. Additionally, climate change is expected to increase climate variability, further complicating the creation and reliability of climate knowledge and thereby broadening the existing ‘valley of death’ between climate knowledge producers and users [3]. This lack of clarity has resulted in a broad and varying definition of what falls under CIS, thereby creating confusion and fragmentation both in the literature and in practice [4]. When attempting to classify CIS, a fundamental variable to look at is the engagement level of users in the production of climate information. Vedeld et al. [5] classify engagement levels in climate services by distinguishing between information provision, dialogue-based engagement, co-production and co-creation. While the literature agrees on the need for increased tailoring and user engagement in the form of co-production and/or co-creation ([6,7]), real-life examples of these forms of user engagement remain limited [8]. Experience with conceptualization and implementation of higher forms of user engagement is therefore currently limited and definitions of co-production and co-creation remain dependent on different authors' interpretations ([[9], [10], [11]]).

Here we address this knowledge gap by looking at the conceptualization and implementation of user-engagement in three recent projects, which serve as case studies. By doing this, this study contributes to defining clear and shared definitions of co-production and co-creation, both in conceptualization as well as practical implementation. When doing this, the focus will be particularly on the understanding and perceptions of user engagement by service providers. Although users should play the main role in user engagement activities, it is of high importance that service providers have a clear understanding of what they are doing before such activities can be successfully implemented. Only when user engagement definitions are well understood and shared by researchers and practitioners alike, can these contribute to improving how users are actively involved in CIS, thereby also developing capacity-building programs for these users.

## 2 Theory

### 2.1 User engagement and its classification

User engagement has a long tradition and is a known concept also outside the climate services sector, among others product-service systems and sustainable product and service innovation ([12,13]), and both in the public and private sectors [14]. Engagement can be seen as ‘a dimension of usability and is influenced by users’ first impression of an application and the enjoyment they derive from using it’ ([15], p.938). When it comes to climate services, user engagement can be defined as all attempts to ‘achieve greater connection to stakeholders’ needs by integrating participatory activities into the climate services development agenda’ and are seen as a key step towards implementing CIS effectively ([16], p.1).

Multiple attempts have been carried out in the literature to classify user engagement in climate services [17], while others have explored required steps for the selection and engagement of users [18]. In our research we look at the user engagement levels implemented after user selection has been completed. We have done this by classifying user engagement types under the interpretation by Vedeld et al. [5], which distinguishes four levels of user engagement, namely information provision, dialogue-based engagement, co-production and co-creation. The framework of Vedeld et al. [5] was selected for this study for two main reasons: 1) it allows to clearly distinguish between co-production and co-creation, and 2) it provides clear guidelines on what to base this distinction on in such a way that allows for easy comparison to real-life case studies.

**Fig. 1:**
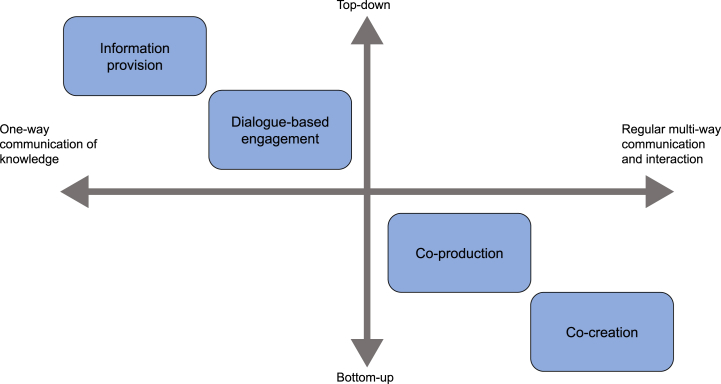
Matrix to classify the user engagement level in climate services (adapted from Vedeld et al. [5]).

Fig. 1 positions the four forms of user engagement in a matrix and classifies them on the basis of 1) top-down versus bottom-up services’ design, and 2) one-way versus mutual communication and interactions. The four forms are further elaborated below.

#### 2.1.1 Information provision and dialogue-based engagement

Information provision and dialogue-based engagement fall under the definition of low forms of citizens’ participation. Users can access information through websites and brochures, give opinions, sign petitions or take part in consultations or and/or seminars ([19,20]). While they both involve mainly web-based, one-way transfer of information and a top-down method, communication and interaction are further developed through dialogue-enhancement mechanisms e.g. feedback surveys or trainings [5].

#### 2.1.2 Co-production and co-creation

Definitions of both co-production and co-creation remain highly debated ([[9], [10], [11]]). The two terms are often used interchangeably within the climate services literature and practice, and many definitions of co-production and co-creation describe them as almost synonymous. In this research, we define co-production and co-creation as two distinct user engagement levels and do so by drawing from the interpretation by Torfing et al. [21] from public administration literature. Under these definitions, co-production is ‘the interactive process through which the providers and users of public services apply their different resources and capabilities in its production and delivery’ (p.8). Co-creation is ‘a process through which two or more public and private actors attempt to solve a shared problem, challenge or task through a constructive exchange of different kinds of knowledge, resources, competencies and ideas that enhance the production of public value’ (p.9). Our definition of co-production therefore distinguishes itself from other interpretations within the climate services field, like those by Vincent et al. [22] or Meadow et al. [23], where it is represented as close to synonymous to co-creation. Nevertheless, we find this distinction to be necessary to define distinct user engagement levels to be followed depending on one's goals and contexts when developing climate services. A list of alternative definitions of co-production and co-creation that can be found in the literature is presented in the supplementary data.

### 2.2 Characterizing the implementation of co-production and co-creation

The three case studies are studied based on the elements of institutional design as identified in the implementation framework of Vedeld et al. [5], illustrated in Table 1.

**Table 1:** Differentiation between co-production and co-creation based on the institutional design components (adapted from Vedeld et al. [5],).

Institutional design component	Co-production	Co-creation
*Designs of arenas of interaction and collaboration*	Co-design of services Multi-way communication	Co-design & co-implementation of services Frequent multi-way communication
*Participation mechanisms*	Face-to-face interactions Broad set of tools and channels of communication	Face-to-face interactions Intense interaction among multiple actors (workshops + interface with social media)
*Degree to which the services are decentralized and engage in on-site activities*	Include some level of on-site extension support and follow-up on the ground	Inclusion of practice-based knowledge Social learning at local level

When looking at the three institutional design components (Table 1), differences between co-production and co-creation can be specified. Co-creation involves higher user engagement through co-implementation, regular multi-way communication, workshops and/or social media interfaces and the inclusion of local/traditional knowledge [5].

The aim of this study is to better grasp the conceptualization as well as actual implementation of higher levels of user-engagement, as these have been less explored in literature and practice [19]. The following mainly focusses on co-production and co-creation. When characterizing conceptualizations of user engagement by providers, a distinction is made between implicit and explicit conceptualizations. With *explicit* conceptualization it is meant the terms that service providers use to describe the user engagement that was implemented in their respective projects. *Implicit* conceptualizations are instead deduced from how providers position the user engagement within the matrix in Fig. 1. Distinguishing between implicit and explicit conceptualizations provides an additional layer to this study, as it can highlight different interpretations of user engagement types between different service providers. Looking at the *implementation* of user engagement provides additional insights in how the theoretical understanding of providers was translated into action during the CIS implementation and can bring attention to similarities in the implementation of CIS that have been conceptualized differently.

### 2.3 Study aims

The aim of this study is achieved by answering the main research question regarding what are the conceptualizations and implementations of user engagement by CIS providers for three different types of CIS.

This is achieved by determining (1) what are the providers’ implicit and explicit conceptualizations of user engagement in their services on the basis of the different user engagement levels in Fig. 1. Then, (2) the user engagement that was actually implemented in the three case studies is characterized based on the institutional design components formulated by Vedeld et al. [5]. Finally, (3) the main differences and similarities between (implicit and explicit) conceptualizations of user engagement and its implementation are pointed out in the three case studies.

## 3 Case studies selection

In this research, a cross-case design research approach is undertaken, by analyzing three case studies. This approach allows us to look at three similar real-life CIS examples and to compare their results so to allow us to draw conclusions which can be applied to the broader CIS field [24]. The case studies were selected so to have enough in common to be compared with each other, but to differ enough to represent the broader climate services domain. Consequently, these were chosen based on aim, design, providers, sector and temporal scale. Only CIS aiming for climate adaptation were looked at, as these are better represented in climate services and related literature. Subsequently, CIS were looked for where some level of user engagement could be expected. This expectation is based on the aim of the service, the output and/or the way in which user engagement is described in their context. Finally, case studies were selected which differ in providers, sector and temporal scale in which the information is provided.

The following case studies were chosen: the WaterApps project by Wageningen University and Research, the Dutch company Buienradar and the EU Copernicus project WaterSIS (Table 2).

**Table 2:** List of three case studies of this research and their characteristics. All three CIS aim at climate adaptation rather than mitigation and claim to involve some level of user engagement.

CIS	Providers	Sector	Temporal scale
WaterApps	Wageningen University & Research Centre (WUR)	Agriculture	Short-to-medium term weather forecasts, seasonal forecasts
Buienradar	Private sector	General population	Short-term weather forecasts
WaterSIS	(Inter-)national providers (EU & SMHI)	Water sector	Seasonal forecasts, long-term climate forecasts

### 3.1 WaterApps

WaterApps (2016–2021) was a research project carried out by Wageningen University & Research in the context of the Urbanizing Deltas of the World Program. The project aimed to develop water and climate information services for and with farmers in two locations, Accra (Ghana) and Khulna (Bangladesh), thereby improving both water and food security [25]. WaterApps actively engaged with users (smallholder farmers, agricultural extension officers and governmental officials) to co-design services through intensive collaboration. The project has resulted in the creation of a FarmerSupport APP prototype and design principles for future development of participatory Climate Services for smallholder farmers [26].

### 3.2 Buienradar

Buienradar is a private Dutch company founded in 2006 that provides both real-time weather information and short-time weather forecast at national and local scale ([27,28]). The CIS is provided by the private sector to the general population of the Netherlands. The service is accessible through the Buienradar website and mobile app. In addition, Buienradar is present on multiple social media platforms and its website includes a blog [29]. Its large social media presence and the constant interaction with users there can be seen as indicators of user engagement in the CIS.

### 3.3 WaterSIS

WaterSIS is an operational service for the Water Sector within the Copernicus Climate Change Service (C3S) of the European Union ([30,31]). C3S is a service with the aim of providing information to facilitate adaptation and mitigation policies at the European level in different sectors [31]. WaterSIS provides such information and support for the water sector specifically [30]. WaterSIS informs water experts, managers and decision-makers to facilitate adaptation and mitigation [30]. The project included one webinar with potential users and the activities carried out during the workshop were described as co-design, which were considered as elements of user engagement. While its data, design and operational systems have been developed within the boundaries of C3S, WaterSIS itself is being developed by the Swedish Meteorological & Hydrological Institute (SMHI), which is therefore also its main provider [30]. The service will include both long- and short-term climate projections, depending on the different user groups making use of the service [30].

## 4 Material and methods

A rich picture from the three cases was developed by combining various data sources to answer the three research objectives (Table 3, Table 4). While semi-structured interviews were collected for all three case studies, other qualitative data collection methods were used for individual case studies depending on the project characteristics and related activities and outputs. The interview guides are shown in supplementary data.

**Table 3:** List of data collection methods implied for each of the three sub-objectives. The last sub-question was answered by combining the answers to all previous sub-questions.

Sub-objectives	Data collection methods
*(1) Implicit and explicit conceptualizations of user engagement by CIS providers*	8 individual semi-structured interviews with CIS providers + 1 interview with 2 CIS providers + 6 academic publications + documentation from one webinar
*(2) Characterization of user engagement during implementation CIS based on institutional design components*	8 individual semi-structured interviews with CIS providers + 1 interview with 2 CIS providers + documentations (2 for WaterApps and 1 for WaterSIS), 6 publications and observation of one webinar
*(3) Difference and similarities between case studies*	Answers to the previous sub-question are brought together

**Table 4:** List of data collection methods implied for each of the three case studies. The last sub-question was answered by combining the answers to all previous sub-questions.

Case study	Data collection method
WaterApps	4 individual semi-structured interviews with CIS providers + academic publications
Buienradar	2 individual semi-structured interviews with CIS providers +1 interview with 2 CIS providers
WaterSIS	2 individual semi-structured interviews with CIS providers + observation of 1 webinar & related documentation

### 4.1 Implicit and explicit conceptualizations of user engagement by CIS providers

Information about implicit and explicit conceptualizations was collected through nine semi-structured interviews with ten service providers. All interviewees were involved with the development and/or implementation of one of the case studies. The selection of the interviewees was carried out through a snowball sampling method. The interviews were all semi-structured so to allow the collected information to be comparable with each other, but at the same time to leave space for alternative questions when necessary [32]. Information from the interviews was supplemented with academic publications in the WaterApps case, and documentation from the webinar for prospective users (in the WaterSIS case). The interviews were used to collect information about how CIS providers implicitly and explicitly conceptualized user engagement within the service in question. Explicit conceptualizations were collected by asking providers how they would define user engagement in their CIS. Implicit conceptualization were deduced by asking providers to position the service within in an empty version of the user-engagement matrix in Fig. 1. The resulting matrix for each interviewee were averaged so to obtain one filled-in matrix for each case study. These matrixes were then used to qualitatively compare the case studies with each other. The interviewer introduced the focus of the interviews as related to user engagement in climate services and did not mention the concepts of co-production, co-creation or any other user engagement form, so not to influence the providers’ responses. The terms were only introduced to the providers at the last question, when providers were asked whether they were aware of the terms co-production and co-creation and whether these had been used to describe user engagement in the projects they were involved in. The concepts of implicit and explicit conceptualizations were not mentioned to the interviewees but were only used in the data analysis.

### 4.2 Implemented user engagement and its characterization

A combination of sources was used to collect information about implemented user engagement in the three case studies (Table 3). For this purpose, questions regarding the (perceived) role of users in the design of the service were asked. In addition, interviewees were asked about the role of capacity building, user guidance and practiced-based/local/traditional knowledge in the services. Finally, specific questions regarding the translation of the service aims in practice, the attitude of users towards these activities and their impact were asked.

The information from the interviews was supplemented with internal and external documentation and through the WaterSIS webinar. Documentation from both WaterSIS and WaterApps was used to collect information about the initial aims of the projects in terms of user engagement and in how far these ended up being implemented. For WaterSIS, the prospective users' webinar allowed to observe user engagement while it was happening. The collected information was then compared with Vedeld et al.‘s [5] institutional design components (Table 1) to determine whether the implemented user engagement could be better characterized as co-production, co-creation or neither.

All the collected data was analyzed in a qualitative manner by means of deductive coding in Atlas.ti. Deductive coding was done using the theoretical framework of this research, namely the three institutional design components of the co-creation framework by Vedeld et al. [5]. This resulted in the identification of 16 codes, shown in supplementary data.

## 5 Implicit conceptualizations of user engagement by providers

**Fig. 2:**
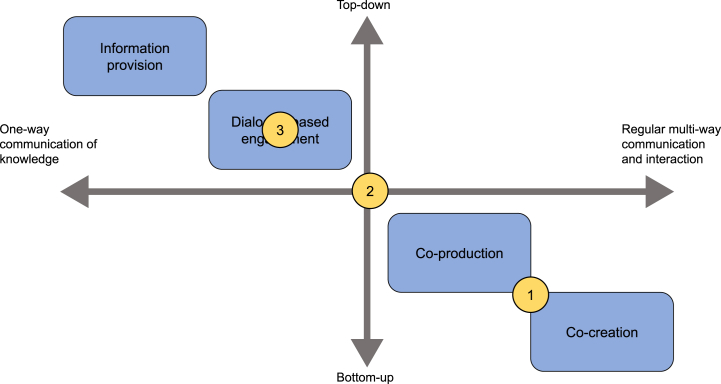
Comparison of the implicit conceptualizations of user engagement in the three climate services (WaterApps: 1, Buienradar:2, WaterSIS: 3), as defined by the services' providers. The positioning of each case study is based on the average of the implicit conceptualization of the different providers who were interviewed.

In this section the implicit conceptualizations by the services’ providers are shown. Fig. 2 shows that providers implicitly characterized user engagement in their services differently from each other.

On average, WaterApps providers characterized the service as being in between co-production and co-creation, whereas Buienradar was characterized as fitting in between dialogue-based engagement and co-production. WaterSIS was instead characterized as including elements of information provision, dialogue-based engagement and co-production. Different providers of each individual case study often disagreed on their implicit conceptualizations. These different interpretations are elaborated per case study below.

### 5.1 WaterApps

WaterApps interviewees conceptualize user engagement as either co-production, co-creation or somewhere in between when asked to position the service within the matrix. All interviewees agreed that WaterApps made use of regular multi-way communication and interaction. Different project activities allowed for communication and interaction amongst farmers, between them and providers, but also with agricultural extension officers. Most clear examples of this are a WhatsApp group for farmers to communicate their measurements and exchange weather information and forecasts and the Weather Clubs, a platform for farmers to discuss and develop skills in weather forecasting and its interpretation.

Disagreement was found on the extent to which providers defined WaterApps as top-down or bottom-up. Most interviewees agreed that while WaterApps is best defined as bottom-up, the project did involve some top-down elements. These elements included how the providers approached the villages as researchers with specific aims which were not negotiable and how the providers implied their own ICT knowledge to create the app, which the farmers would not have been able to do themselves. Nevertheless, this was not seen as an obstacle for defining the project as bottom-up, as contributions from farmers were encouraged and actively stimulated. Two providers did though position various elements of the project differently. One indeed mentioned how the process that led to the WaterApps project had a clear top-down approach, which is again related to the context of the research, its objectives, the goals of different PhDs and the management plan. The project implementation should instead be defined as more bottom-up due to the large engagement of farmers in the practical activities. Another provider instead classified the WaterApps project as being completely bottom-up. The main reason for this was found in how the farmers were involved and engaged already from the design stage of the CIS. The interviewee presented this as being largely different than most climate services, where the information is directly provided by the national meteorological department. WaterApps then included the bottom-up element of bringing the meteorological department to the field and allowing them to listen to the farmers and build relationships with them so to better tailor the weather information to their needs.

### 5.2 Buienradar

Implicitly, Buienradar providers conceptualize user engagement in the service as being either dialogue-based engagement, co-production or just in between the two. All interviewees agreed on how information provision in Buienradar had an important top-down component. Two providers defined the design as being in between top-down and bottom-up and identified the large number of users as the reason for the top-down elements. Such a large user interface was therefore seen as a practical obstacle to the involvement of users in all decisions. Another study participant instead identified specific features such as cookie walls as top-down elements, which are dictated by profit requirements of the company and unrelated to users' needs. These features are balanced out by bottom-up features which are developed based on users’ information needs and at times specific requests. Finally, one study participant indicated Buienradar as being prevalently top-down due to the nature of the information that is provided by the service, which requires specific technical skills.

Providers instead disagreed when classifying Buienradar in terms of communication and interaction. Providers described Buienradar as involving one- and multiple-way communication and interaction. While weather information is provided in a one-way direction from Buienradar to its users, user interaction with the provided information is frequent and stimulated by Buienradar itself. One provider instead believed that communication and interaction could be defined as being multi-way. Another provider suggested a distinction between the products of Buienradar, like the website and the app, and the company's online presence. When purely looking at the company's products, one-way communication remains dominant. Nevertheless, on the social media platforms, interaction is more prevalent.

### 5.3 WaterSIS

The two WaterSIS providers implicitly conceptualize user engagement in the service as either being in between information provision and dialogue-based engagement or in between dialogue-based engagement and co-production. When looking at the design of the WaterSIS project, the providers disagree. One provider described the service as being more top-down rather than bottom-up, while another identified some bottom-up components. In particular, the process of developing the indicators to be shown by the WaterSIS was described as bottom-up. In terms of communication, one provider defined WaterSIS as being predominantly one-way, with one user webinar as only exception. Another provider instead believed the communication to be multi-way and justified it with both the webinar and the positioning of WaterSIS within the broader C3S context.

## 6 Explicit conceptualizations of user engagement by providers

In the following section, the explicit conceptualizations of user engagement by the services’ providers are presented, with the aim of comparing these with the implicit conceptualizations just described.

### 6.1 WaterApps

All WaterApps providers explicitly conceptualized user engagement in WaterApps as co-production. They also specifically highlighted how co-production was a better fit to describe user engagement in the service than co-creation.

### 6.2 Buienradar

Two providers explicitly conceptualized user engagement in Buienradar as co-creation, by defining it as a process where the idea for the service is developed, or created, together with the users, but where the weather information is made by the providers only. Two other providers were not familiar with the user engagement terms used here. Instead, they described user engagement in Buienradar as an interactive, full and mixed process that results in a user-focused product. In general, all providers mentioned how neither co-production nor co-creation are regularly used when discussing user engagement within Buienradar. Terms such as interaction, (user) surveys and user research are instead used regularly.

### 6.3 WaterSIS

Explicitly, providers conceptualized user engagement in WaterSIS as co-design. During the WaterSIS users workshop, co-design was defined as the practice of identifying the needs or requests of the users in terms of application and guidance and training. The objective of such an approach is then to bridge the gap between the WaterSIS developers and the experts and to identify potential showcases to be used to assess the service that has been created. One provider highlighted how this is merely the definition of co-design in the specific WaterSIS context and how different definitions of the term exist. The same provider also mentioned how in a general sense the activities of WaterSIS do not truly involve co-design, which would require much more than feedback on application design. The activities were still defined as co-design in the webinar context due to the familiarity that users have with the term. When looking at the implemented activities, the provider simply defined it with the overarching terms user engagement or participatory processes creating a community of users around WaterSIS.

## 7 Characterization of implemented user engagement in the three CIS

Fig. 2 shows that when looking at the user engagement activities implemented in the projects, these differ between the three case studies.

On the basis of our analysis, WaterApps was shown to have implemented co-creation. The other two case studies showed lower implemented user engagement, with Buienradar showing characteristics of dialogue-based engagement (with individual elements of co-production and even co-creation) and WaterSIS those of dialogue-based engagement or lower. Justification of at which user engagement level each case study implementation activities fall is described per case study and per institutional design component below.

### 7.1 Design of arenas of interaction and collaboration

#### 7.1.1 WaterApps

WaterApps can be characterized as co-creation when looking at the *design of arenas of interaction and collaboration*, due to the presence of elements of both *co-design* and *co-implementation* and *regular multi-way communication*. What can be classified as *co-design* and *co-implementation* can be rather blurred for this project. When the whole research process is taken into consideration, the design stage is the development of the research proposal and related objectives, while the implementation starts from the moment when the activities in the field took place. Under this interpretation, the process involved *co-implementation* with the farmers, as these have been involved once field work started, but not before. *Co-design* was therefore limited, as all objectives and decisions of the whole research were determined in the proposal stage, preceding the involvement of the farmers. *Multi-way communication* was found to take place frequently. Already during the development phase, information about farmer's feedback on the prototype was exchanged frequently. During implementation, the interaction took place face-to-face once a week and daily online.

#### 7.1.2 Buienradar

Table 4 shows how Buienradar is characterized by implementing some elements of co-production. The standard Buienradar design involves the creation of weather forecast based on different weather models. This information is then presented in a simplified form to users through the Buienradar app and website. Users are involved in the design (*co-design*) phase but not in the implementation phase (*co-implementation*) of the service and *multi-way interaction* is present only to some extent. An example of *co-design* is the recent rebuild of the Buienradar app, which followed discussions with users to determine desired improvements and functionalities. The newly introduced 8-h forecast was also first suggested by users through consistent feedback and surveys. Feedback on the forecast or specific app functionalities is continuously provided by users through different platforms. This results in constant development in the form of new campaigns. In terms of *co-implementation*, one example could be found. If users find errors in a forecast and share this online, the information can sometimes be used to improve radar's accuracy. Buienradar continuously provides weather information to its users through different platforms and in different formats. At the same time, users can always provide feedback to Buienradar. This can happen either through the app, the website, the app store or different social media platforms. Actual *multi-way communication* only happens in specific settings, for example when users leave their contact information in the feedback on the app or when Buienradar contacts users who have shown interest in the past to carry out in-depth conversations about individual features or issues. Consequently, while a limited number of users have participated in interviews or focus groups, for the vast majority of users communication is one-sided.

#### 7.1.3 WaterSIS

Despite naming user engagement in the service as co-design, in WaterSIS little to no examples of *co-design* as understood by Vedeld et al. [5] could be identified. This was intentional and determined by the inherent design of the service. WaterSIS is one of many projects hosted on the C3S platform and must therefore follow its functionality. SMHI providers did not have complete freedom in the development of the service, which was reflected in the amount of user input that could be included. While user feedback was looked for, some aspects of the service had to fit within the broader C3S functionality. This is not to say that *co-design* had no place in the project, but it did not take place in the specific context of the WaterSIS. Instead, the experience of previous projects, like SWICCA, Edge and GLORIOUS have been mentioned as the basis for the current WaterSIS design. *Co-implementation* instead took place in the form of one webinar, user surveys and in showcases. The objectives of the webinar included collecting feedback on the usability of pilot WaterSIS website and investigating users’ needs. In practice, users were asked to provide feedback on the design of the WaterSIS website, on the data visualization methods (e.g. structure and design of graphs, types of scenarios) and on the required user guidance.

Communication in WaterSIS can be characterized as one-way and partly *multi-way*. Elements of *multi-way communication* took place sporadically, and only included few users. By the time of this research, communication between WaterSIS providers and users took place during the webinar and through surveys. *Multi-way communication* was thus not frequent, as in the whole project only one webinar has taken place. All webinar participants were experts and/or scientists, which resulted in the collection of mainly research-oriented feedback. This excluded policymakers, one of the WaterSIS stakeholder groups, from the user engagement process so far. The inclusion of a very limited number of users was intentional, as providers aimed to engage a small set of users who already had experience with C3S and/or SWICCA. In this way, the feedback could focus on the developments implemented in the switch from SWICCA to WaterSIS. Thus, as indicated in Fig. 2, WaterSIS user engagement implementation did only to a very limited extent reflect co-production or co-creation elements. User engagement implementation can better be characterized as lower level user engagement, associated with sporadic one-way communication and lack of co-design or co-implementation.

### 7.2 Participation mechanisms

#### 7.2.1 WaterApps

The Participation mechanisms implemented in WaterApps relate to co-creation, as the CIS activities took place *on-site* and a combination of different *face-to-face* and *online communication channels* have been implied to bring together researchers, farmers and local extension officers. Information sharing and exchange within the WaterApps was stimulated from providers to farmers and the other way around, but also between farmers and between farmers, provider, and local extension officers. This interaction was made possible by the development of long-lasting relationships, which were the focus of preliminary research. Later in the project, group discussions also stimulated interaction between farmers. The regular and frequent communication has been taking place among multiple platforms, namely the farmers’ Weather Clubs, WhatsApp group for farmers and a Facebook group for local extension officers.

#### 7.2.2 Buienradar

Buienradar *participation mechanisms* mainly fit within co-production, due to some elements of *face-to-face interactions* and *communication* taking place through a *broad set of tools and channels*. Communication between Buienradar and its users takes place along *different tools and channels of communication*, including social media (Instagram, Twitter and Facebook), App stores, the Buienradar app and the Buienradar blog. A restricted number of users also engage in *face-to-face interactions* through interviews and brainstorming sessions during development of new features or functionalities. While interaction between Buienradar and its users is frequent, this is not *intense*, as comments below Buienradar social media posts rarely result in conversations. Interaction through social media mostly involves weather presenters posting short summaries of the forecast and the users providing feedback on the accuracy of such forecasts. Interaction is also actively stimulated by Buienradar through e.g. the creation of polls or challenges or the request to share photos of the weather.

#### 7.2.3 WaterSIS

The participation design element in WaterSIS could not be characterized by any of the elements representing co-production or co-creation (Fig. 2). No *face-to-face interactions* have taken place within the WaterSIS context. Communication took place exclusively online through one webinar and surveys. The *tools and channel of communication* are therefore not broad. In addition, the interaction was not intense, and the number of actors involved was small.

However, WaterSIS was the only case study to be affected by the COVID-19 pandemic. Interaction with the users was supposed to be continuous and to start much earlier in the project, but due to both the pandemic and some delays this only started in November 2020 in the form of surveys. In addition, the webinars were supposed to be three in total and would have taken place in person. The pandemic stopped users from travelling and meeting in person, therefore only one online webinar took place. In addition, at least one additional webinar is planned for after the new application has been launched. This will have the aim of presenting the application to the broader C3S user network. Next to this, the providers are currently looking for potential showcases, which would show how the data provided through the service can be used by real-life examples.

### 7.3 Degree to which the services are decentralized and engage in on-site activities

#### 7.3.1 WaterApps

WaterApps again falls under the definition of co-creation for the last indicator. Some differences can be seen between the fieldwork in Ghana and Bangladesh. In Ghana, the WaterApps team produced their own information for the WeatherApp, where the scientific and local weather forecasting are shown next to each other. In Bangladesh, the focus instead laid on improving the quality and impact of the existing one. The focus then switched from the inclusion of *local* weather forecast *knowledge* towards more *social learning at the local level* in the form of *capacity-building* towards scientific weather forecasts and its interpretation. In general, the great majority of the WaterApps project took place *on site* and the presence of the personnel on site was identified by the providers as a key element of the project, as they set the basis for creating a network of trust.

*Practice-based knowledge* is included in WaterApps in the form of local weather forecasting based on ecological indicators used by local farmers. Some elements of inclusion of local knowledge can be identified in both research sites, but more largely in Ghana. The second requirement for co-creation, *social learning at the local level*, can also be found in both WaterApps settings. In the Bangladesh case, this played a key role throughout the project, with activities aiming at developing capacity building of farmers. These activities included providing farmers with insights on their own information requirements and training to understand and interpret multiple formats of climate information. The *capacity building* of local extension officers was also positively influenced by the WaterApps project. By sharing the weather forecast on a Facebook group, extension officers were introduced to the importance of climate adaptation to their operation in advising farmers.

#### 7.3.2 Buienradar

Table 4 shows how Buienradar only includes one individual element of co-creation for the last indicator and can for the rest mostly be characterized as a lower user engagement form. Buienradar cannot score well for this final institutional design component, as this would require at least some level of on-site extension support and follow-up on the ground, which is less relevant for the company. Buienradar is indeed a Dutch company that only provides information on the weather in the Netherlands, therefore the location of the providers, the produced information and the users is the same. Buienradar does though include elements of *social learning at the local level* through the creation of blog posts. These are to educate Buienradar user on specific weather phenomena. In addition, blog posts also present models' uncertainties and justify the unpredictability of specific weather events. This has the potential of improving users’ understanding of the weather system and of the functioning of Buienradar, therefore leading to *social learning*. *Practice-based knowledge* can here be better understood as citizen science or user-generated content. This is not yet incorporated in the service because the team could not make good use of this knowledge. First, while some smartphones can already measure temperature, humidity and air pressure, the measurements are not yet accurate enough, which makes the collected information not useful for forecasting. Second, the extremely large user base of the service can potentially result in false claims which would influence the forecasting.

#### 7.3.3 WaterSIS

WaterSIS was found not to include any type of *on-site extension* support, nor inclusion of *practice-based knowledge* or *social learning at the local level*. While the lack of on-site extension support could be related to the COVID-19 pandemic, the nature of the service in itself does not include any *practice-based knowledge*. *Social learning at the local level* might take place thanks to user guidance. Nevertheless, this cannot be confirmed at this point in the project, as the user guidelines are still being developed at the moment and are not available yet.

### 7.4 Case study comparison

When looking at the implicit and explicit conceptualizations of user engagement by providers and the implemented user engagement in the three case studies, a number of similarities and differences can be identified (see Table 5) (see Table 6).

**Table 5:**
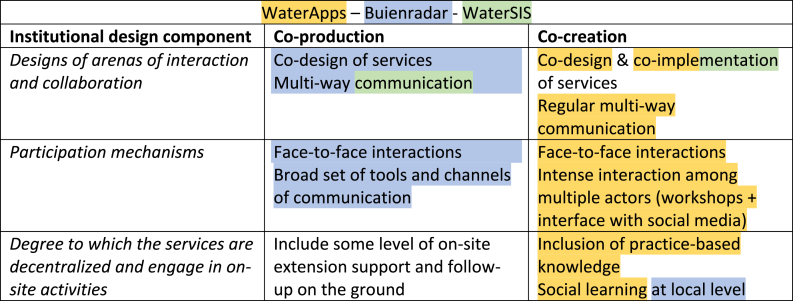
Characterization of the implemented user engagement in the three case studies based on the institutional design components developed by Vedeld et al. [5], as compiled from the providers’ interviews and related documentation. All elements highlighted in orange were identified within WaterApps activities, those in blue in Buienradar activities and those in green in WaterSIS activities. When one component is highlighted in two different colors, this means that both case studies were found to include this element in their user engagement implementation.

**Table 6:** Results of the analysis of implicit and explicit conceptualizations of user engagement by CIS providers and implemented user engagement for the three case studies of this research.

CIS	Implicit conceptualization by providers	Explicitly conceptualization by providers	Implemented user engagement
**WaterApps**	Co-production/Co-creation	Co-production	Co-creation
**Buienradar**	Dialogue-based engagement/Co-production	Co-creation *(when aware of terminology)*	Dialogue-based engagement/Co-production *(plus one element of co-creation)*
**WaterSIS**	Information provision/Dialogue-based engagement/Co-production	Co-design	Dialogue-based engagement *(plus one element of co-production and one of co-creation)*

One similarity is that in neither of the case studies there was a complete agreement on the implicit conceptualization of user engagement between all providers. WaterApps providers agreed on the level of communication and interaction of the service but disagreed on conceptualizing its approach (top-down versus bottom-up). WaterSIS showed the most differences in implicit conceptualizations by providers. This was the result of disagreements between both the approach taken by the service and the amount of communication and interaction with the users. These results suggest that not all providers shared a vision on the nature of the user engagement that they had been carrying out within the service.

In addition, in none of the case studies there was a complete overlap between implicit and explicit conceptualizations of user engagement by the providers. WaterApps was the service where the most overlap was found, with implicit conceptualizations as in between co-production and co-creation and explicit as co-production. In Buienradar, one provider conceptualized the user engagement as co-creation, while the others described it as an interactive, full and mixed process that results in a user-focused product.

In general, the similarities between conceptualizations and the implemented user engagement varied per case study. WaterApps showed the highest similarities, as both implicit conceptualization and the implemented user engagement could be mostly characterized as co-production. The only difference could be found in the explicit conceptualization of user engagement, which was co-production. This shows that WaterApps providers had a shared understanding of how they conceptualized user engagement, and that this conceptualization was also in line with the user engagement that has been implemented in the project. In the Buienradar case there appeared to be once again an agreement between the implicit conceptualization by providers and the implementation of user engagement, both falling in between dialogue-based engagement and co-production. Explicit conceptualization instead differed and corresponded to co-creation when this term was known by providers. In WaterSIS, implicit conceptualizations have been shown to differ per provider. The implemented user engagement could be characterized as dialogue-based engagement, which is in line with one of the two implicit conceptualizations.

Finally, in all three case studies elements of multiple user-engagement levels could be identified in their implemented user engagement. WaterApps can be identified as including some elements of co-production and some (the majority) of co-creation. For Buienradar, the implemented user engagement can be described as in between dialogue-based engagement and co-production, also including one specific element of co-creation. WaterSIS was instead found to predominantly include low levels of user engagement, with the webinar as an important exception.

## 8 Discussion and conclusion

### 8.1 A theoretical issue with concrete impact

The results of this research suggest that the current lack of clarity regarding user engagement in CIS is a theoretical issue, but one that has clear impacts in practice. Although high forms of user engagement can still be identified in the case studies, the confusion is apparent in providers’ both implicit and explicit conceptualizations of user engagement. We argue that a shared understanding of the terminology is of fundamental importance for a climate service to be successful, especially considering the novelty of this field of study [9] and its interdisciplinarity. In particular, the current lack of clarity runs the risk of further complicating efforts to evaluate and compare different services, which is already challenging [33]. In an interdisciplinary field, it is even more likely for different disciplines to have different understandings of concepts and to value different ways of working. For this reason, making these different understandings of user engagement concepts and values explicit, and thereby creating a shared understanding within the context of a specifical CIS, is even more important.

### 8.2 Complexity as key to a shared understanding

This study suggests that one climate service can be characterized by multiple forms of user engagement. For example, users can be very involved in the CIS implementation (co-creation), while most communication remains one-sided (dialogue-based). This raises the question of how to describe the user engagement of a particular service in such situations. One solution could be to simply characterize implemented user engagement of a service with that as indicated by most of its characteristics. If one service has for example most of the characteristics of co-production and individual elements of co-creation, this should be defined as involving co-production. While such an approach is straightforward, it might result in excessive simplification. Based on our results, a different approach is proposed, which highlights the different elements of user engagement in CIS. CIS are complex and include multiple elements and stages, which is also the reason why their definition and classification is still so discussed [1]. When characterizing user engagement in CIS, their complexity should therefore be embraced, so to explore how different stages and elements of a service can be characterized as involving multiple different levels of user engagement. This is a similar approach to that of Cepiku et al. [14], who looking at the public sector identify multiple analytical components within key dimension of co-production, namely general context, antecedents, management and outcomes. Within the CIS literature specifically, this is in line with the approach taken by Bremer et al. [9], who propose a multi-faceted understanding of co-production of climate services instead of the current narrowly-framed interpretations. Steuri et al. [34] further support this by finding that user engagement styles tend to change throughout the different stages of a climate service. Our conclusions then bring these different concepts together by proposing a multi-faceted understanding of user engagement.

### 8.3 Limitations and shortcomings

While a number of relevant messages can be taken out of this research, it is also important to recognize its shortcomings. Main points of attention are the data collection and analysis, theoretical framework used and the differences between case studies.

Firstly, it is imperative to recognize that in this research user engagement has been studied from the perspective of climate services providers only. While their perspective is still important for further understanding the complex environment within which climate services are developed and the current state of the field [34], as explored by Terrado et al. [35], this has implications for our results.

First, this is noticeable when looking at both implicit and explicit conceptualizations of user engagement, as the services here tend to score higher in terms of user engagement level compared with the characterization of the implemented user engagement. This shows how in general service providers conceptualize the user engagement in their activities as higher than in reality. This finding is not surprising. User engagement is more and more seen as a requirement for a successful CIS ([8,36]), which might push service providers to seek user engagement even when this does not benefit the service they offer. This further confirms the current confusion between all existing user engagement levels and different interpretations between different fields, publications and instances. The lack of user perspective in this research also does not easily allow for consideration of power imbalances, which should be accounted for when embarking in a co-production (and consequently also co-creation) process [37]. In addition, the theoretical framework might have influenced the results by favoring one case study. Vedeld et al. [5] originally developed the co-creation framework for agro-met services in India. As the WaterApps project is also an agro-met service that was implemented in two developing countries, it might not be surprising that this case study is showing the most elements of the highest form of user engagement. Specific user-engagement elements of the Buienradar and WaterSIS services might not have been highlighted by the framework due to their characteristics. Nevertheless, it is worth highlighting how this research did not aim to evaluate the three case studies or their implemented user engagement, but rather to highlight the current lack of clarity in the conceptualization and characterization of user engagement in CIS and bring order in this complexity. Therefore, the framework used does not interfere with the aim of the research and the conclusions that can be drawn from it.

It is also important to note how the WaterSIS project differed from the other two in terms of development stage. By the time the interviews were carried out, the development of the WaterSIS project had just been completed and presented to prospective users. This positions the service at a very different stage than WaterApps and Buienradar, which users have already been able to practice with. Several activities have been planned for WaterSIS in the near future, which might influence implemented user engagement. In addition, differently than the rest, the final development stages of the WaterSIS project took place during the COVID-19 pandemic, which strongly limited the potential for face-to-face interaction and communication between and within users. While such limitations should be kept in mind when interpreting the results, these do not influence this study excessively. Nevertheless, we do recommend that future research explores the role of long-distance interactions in user engagement, especially considering the experience learned during the COVID-19 pandemic. When doing so, consideration should be given to the findings by Lemos et al. [38], who argue that, when successful, live webinars and in-person meetings can lead to similar outcomes in terms of one-off efforts to increase usability of climate information. Future research should then explore whether similar results can also be obtained through online engagement of users over long time-periods.

### 8.4 Conclusions

Scholars have highlighted the need for climate services including higher forms of user engagement ([7,36]), like co-production and co-creation. Nevertheless, real-life examples of these forms of user engagement remain limited [8], further contributing to unclarity in definitions, interpretations and implementations of co-production and co-creation. This research brings clarity into the current understandings of the two terms and user engagement in CIS in general by analyzing three case studies, namely Wageningen University's WaterApps project, the company Buienradar and the C3S's WaterSIS project. Implicit and explicit conceptualizations by service providers and implementations of user engagement were explored through the qualitative analysis of semi-structured interviews and documentation and publications. The results of this research bring two main conclusions, both of which improve the current understanding of user-engagement in CIS by its providers and suggest potential ways to bring some clarity in the use of the terminology.

This study confirms the current differences in interpretations of user engagement levels by CIS providers. In addition, we found that co-creation only fully took place in the WaterApps case, where there was a shared understanding of how service providers conceptualized user engagement and where this conceptualization was also in line with the implemented user engagement in the project. This calls for either a change in current user engagement terminology or in the understanding that service providers and users have of the already existing terminology. This would then allow to further implement higher forms of user engagement in climate services and to avoid cases where the implemented user engagement is in fact lower than what service providers believe. Here we suggest that the different user engagement levels within one individual CIS should be highlighted. This would allow the complexity of user engagement in CIS to be embraced by exploring its multi-faceted nature rather than attempting to over-simplify it. Our results contribute to clarifying the theory behind higher forms of user engagement, which can then facilitate practical applications in both climate services, so to accelerate climate mitigation and adaptation.

## Formatting of funding sources

This research did not receive any specific grant from funding agencies in the public, commercial, or not-for-profit sectors.

## Data availability statement

-Has data associated with your study been deposited into a publicly available repository?

No.

- Please select why.

Data will be made available on request.

## CRediT authorship contribution statement

**Valeria Di Fant:** Writing – review & editing, Writing – original draft, Visualization, Methodology, Investigation, Conceptualization. **Maria del Pozo:** Writing – review & editing, Supervision, Conceptualization. **Judith Gulikers:** Writing – review & editing, Supervision, Conceptualization. **Spyros Paparrizos:** Writing – review & editing, Supervision.

## Declaration of competing interest

The authors declare that they have no known competing financial interests or personal relationships that could have appeared to influence the work reported in this paper.
