# Supplementary material for: ‘Conceptualizations and implementation of user engagement in Weather and climate services: A climateservicesproviders’ perspective by Di Fant, V., del Pozo, M., Gulikers, J. and Paparrizos, S
Source: Heliyon. 2023 Dec 12;10(1):e22940. doi: 10.1016/j.heliyon.2023.e22940 (PMC10770519; doi:10.1016/j.heliyon.2023.e22940)
Supplement: Multimedia component 1 [file mmc1.docx]

Appendix A: Existing definitions of co-production and co-creation in the literature

In literature, there is a general agreement that processes of co-production and/or co-creation can result into improved knowledge exchange and collaboration between CIS users and providers, higher implementation of the provided climate knowledge and greater governance relevance of the CIS [[1],[2],[3]]. Nevertheless, definitions of both terms are still highly debated and, in general, authors tend not to distinguish between the two terms, resulting in an understanding of co-production and co-creation as synonymous [[4]]. Alternatively, Bremer et al. [[5]] also highlight how co-production is sometimes referred to as either subsidiary or related to co-creation. Adding to the confusion, the definitions of either term individually are also still in discussion, as for example argued for co-production by Goodess et al. [[6]].

In table A1 below, a restricted number of interpretations of co-production and co-creation in the literature is provided. The list of different definitions is not supposed to be exhaustive, but instead aims to provide examples of confusion and overlaps between different conceptualizations of the terms in the literature.

*Table A1: Overview of definitions of co-production and co-creation by different authors. The two definitions in italics are the ones chosen for this specific research*

| **Reference** | **Definition of co-production** | **Reference** | **Definition of co-creation** |
| --- | --- | --- | --- |
| Norström et al. [[7]], p. 183 | Knowledge co production in sustainability research as the ‘Iterative and collaborative processes involving diverse types of expertise, knowledge and actors to produce context-specific knowledge and pathways towards a sustainable future’ | Ehlen et al. [[9]], p. 630 | ‘a collective process of teamwork across organisations that is creative and geared to generating and developing new products, processes and services, which cause incremental improvements or radical innovations’ |
| Polk [[8]], p.111 | ‘occurs through practitioners and researchers participating in the entire knowledge production process including joint problem formation, knowledge generation and application in both scientific and real word contexts, as well as mutual quality control of scientific rigor, social robustness and effectiveness’ | De Jong, Neulen and Jansma [[10]], p.491 | Can take place when ‘governments and citizens actively collaborate on specific policy issues’ |
| Bremer et al. [[5]], p. 42 | the deliberate, collaborative productdevelopment work between climate scientists, or producers of climate data, and practitioners, or users who require climate information, including potential or even ‘imagined users’’ | Torfing, Sørensen and Røiseland [[4]], p. 9 | ‘a process through which two or more public and private actors attempt to solve a shared problem, challenge or task through a constructive exchange of different kinds of knowledge, resources, competences and ideas that enhance the production of public value (..) either through a continuous improvement of outputs or outcomes or through innovative step-changes that transform the 10 understanding of the problem or task at hand and find new ways of solving it’ |
| Goodess et al. [[6]], p. 4 | ‘a sustained collaborative process between scientists and decision-makers for the production of useful, actionable and socially robust knowledge’ |  |  |
| Torfing, Sørensen and Røiseland [[4]], p. 8 | ‘the interactive process through which the providers and users of public services apply their different resources and capabilities in its production and delivery’ |  |  |

Reference list

1. Alexander, M., Dessai, S., 2019. What can climate services learn from the broader services literature? Climatic Change, 157, 133–149.https://doi.org/10.1007/s10584-019-02388-8
2. Karpouzouglou, T., Zulkafli, Z., Grainger, S., Dewulf, A., Buytaert, W., Hannah, D.M., 2015. Environmental Virtual Observatories (EVOs): Prospects for knowledge co-creation and resilience in the Information Age, Curr. Opin. in Env. Sustain., 18, 40-48. <https://doi.org/10.1016/j.cosust.2015.07.015>
3. Vedeld, T., Mathur, M., Bharti, N., 2019. How can co-creation improve the engagement of farmers in weather and climate services (WCS) in India? Climate Serv., 15, 100103. <https://doi.org/10.1016/j.cliser.2019.100103>
4. Torfing, J., Sørensen, E., Røiseland, A., 2019. Transforming the public sector into an arena for cocreation: Barriers, drivers, benefits and ways forward, Administration & Soc., 51 (5), 795-825. <https://doi.org/10.1177/0095399716680057>
5. Bremer, S., Wardekker, A., Dessai, S., Sobolowski, S., Slaattelid, R., van der Sluijs, J., 2019. Toward a multi-faceted conception of co-production of climate services. Climate Serv., 13, 42-50. <https://doi.org/10.1016/j.cliser.2019.01.003>
6. Goodess, C.M., Troccoli, A., Acton, C., Añel, C.A., Bett, P.E., Brayshaw, D.J., De Felice, M., Dorling, S.R., Dubus, L., Penny, L., Percy, B., Ranchin, T., Thomas, C., Trolliet, M., Wald, L., 2019. Advancing climate services for European renewable energy sector through capacity building and user engagement, Climate Serv., 16, 100139. <https://doi.org/10.1016/j.cliser.2019.100139>
7. Norström, A.V., Cvitanovic, C., Löf , M.F., West, S., Wyborn, C., Balvanera, P., Bednarek, A.T., Bennett, E.M., Biggs, R., de Bremond, A., Campbell, B.M., Canadell, J.G., Carpenter, S.R., Folke, C., Fulton, E.A., Gaffney, O., Gelcich, S., Jouffray, J-B., Leach, M., Le Tissier, M., Martín-López, B., Louder, E., Loutre, M-F., Meadow, A. M., Nagendra, H., Payne, D., Peterson, J.D., Reyers, B., Scholes, R., Ifejika Speranza, C., Spierenburg, M., Stafford-Smith, M., Tengö , M., van der Hel, S., van Putten, I., Österblom, H., 2020. Principles for knowledge co-production in sustainability research, Nature Sustain., 3, 182-190. [[https://doi.org/10.1038/s41893-019-0448-2https://doi.org/10.1038/s41893-019-0448-2](https://doi.org/10.1038/s41893-019-0448-2)](https://doi.org/10.1038/s41893-019-0448-2)
8. Polk, M., 2015 . Transdisciplinary co-production: Designing and testing a transdisciplinary research framework for societal problem solving, Futures, 65, 110-122. <https://doi.org/10.1016/j.futures.2014.11.001>
9. Ehlen, C., van der Klink, M., Stoffers, J., Boshuizen, H., 2017. The co-creation-wheel: A fourdimensional model of collaborative, interorganisational innovation, Eur. J. Train. and Dev., 41 (7), 628-646. 10.1108/EJTD-03-2017-0027
10. De Jong, M., Neulen, S., Jansma, S.R., 2019. Citizens’ intentions to participate in governmental cocreation initiatives, Gov. Inf. Q., 36, 490-500. <https://doi.org/10.1016/j.giq.2019.04.003>

Appendix B: Interview guides

*The interviews took place online using either Skype for Business or Teams and have been recorded. Both via email when first contacting the interviewee and before starting the interview the research was introduced as being about user engagement in CIS without specifying co-production and/or co-creation, so not to influence the providers’ answers. Part 1 and 2 of the interview were asked to all interviewees. Based on their role in the CIS, different questions were asked. When the interviewee indicates to have been involved in the design stage of the CIS, questions from part 3 were asked. When the interviewee was only involved in the implementation of the CIS, then only questions in part 4 were asked. When the interviewee was involved in both design and implementation, all questions were asked. All interviews were concluded with questions from part 5. Questions in bold are the main questions and were asked to all interviewee. Questions in italics are follow-up questions, and were asked or not based on the answers to the main questions.*

**Privacy statement: I would like to record the interview so that I can transcribe it later. The transcript and video will only be used for the aims of my MSc Thesis. Your interview will be anonymized (you will be referred to as one of the CIS-designers), but the name of the CIS won’t. I will also ask whether I can directly quote what the interviewee said or if they would like me t rephrase their answers.**

Part 1: Interviewee personal information (might be filled in by interviewer in advance).

Date:

Name of interviewee:

Educational background:

Employment position:

**How long have you been involved in the project? What is your role in it?**

Part 2: Understanding the CIS.

The aim of these section is to classify the CIS within the framework developed for this research.

CIS name:

Time period (ongoing?):

**1. How would you describe the CIS?**

*-Who came up with the project?*

*-What are is the main aim of the CIS?*

*-Which users and related sectors is it targeting?*

*-What is the spatial and temporal scale of the project?*

*-Which stakeholders are involved in the project?*

*-What type of climate information is produced by the CIS? In what form?*

Part 3: Conceptualization of user engagement.

When introducing this section to the interviewee, this was described as aiming to research user engagement in the CIS.

**2. How would you describe the role of the user in the design of the CIS?**

*-In which stage of the design of the CIS were users involved?*

*-Which activities did you perform with the users?*

*-How often did you perform these activities?*

*-Which type of input did/do users add to the CIS?*

*-What happened with this input?*

**3. How did/does the interaction with the users take place within the CIS?**

*-In what way do users and providers communicate?*

*-Does the communication take place face-to-face or online?*

*-Why have these means of communication been chosen?*

*-How is the communication process organized?*

**4. Which type of user guidance or capacity building did the CIS include, if any?**

*-Are there any elements of the CIS that take place on site? Which ones?*

*-What is the added value of such components?*

**5. How important is users’ own knowledge for the CIS? (Consider practice based/local/traditional knowledge)**

*-What (practice-based/local/traditional) knowledge do users bring to the table?*

*-How does this knowledge compare with the providers’ climate knowledge?*

*-How is this knowledge incorporated into the CIS?*

*-How is this knowledge functional to the CIS?*

Part 4: Operationalization of user engagement

**6. How have the aims of the CIS been translated in practice when implementing the CIS?**

*-How would you describe the role of the user in the activities of the CIS?*

*-Which activities involve the users?*

*-How often do these activities take place?*

*-Does the CIS include the possibility to provide feedback? In what way?*

*-Are all users involved in these activities/feedback moments or only a selected group? Why?*

*-If only selected group: what users are selected? On what basis?*

**7. What was the attitude of the users towards these activities?**

*-Have users been willing to contribute to the CIS? Why do you think that this is the case?*

*-Is answer is no: why not, in your opinion? Are you working on changing this? How?*

**8. What has been the impact of activities with an user component/feedback moments on the CIS?**

*-What would you say were the most important contributions of users to the CIS?*

*-If feedback session: what were the major points of feedback? What happened with the feedback?*

*-Do you feel like user engagement has improved the CIS?*

*-In your work, do you feel like you learned something from the users? What?*

Part 5: Conceptualization of co-production/co-creation

The interviewer received the following empty graph (see fig.B1).


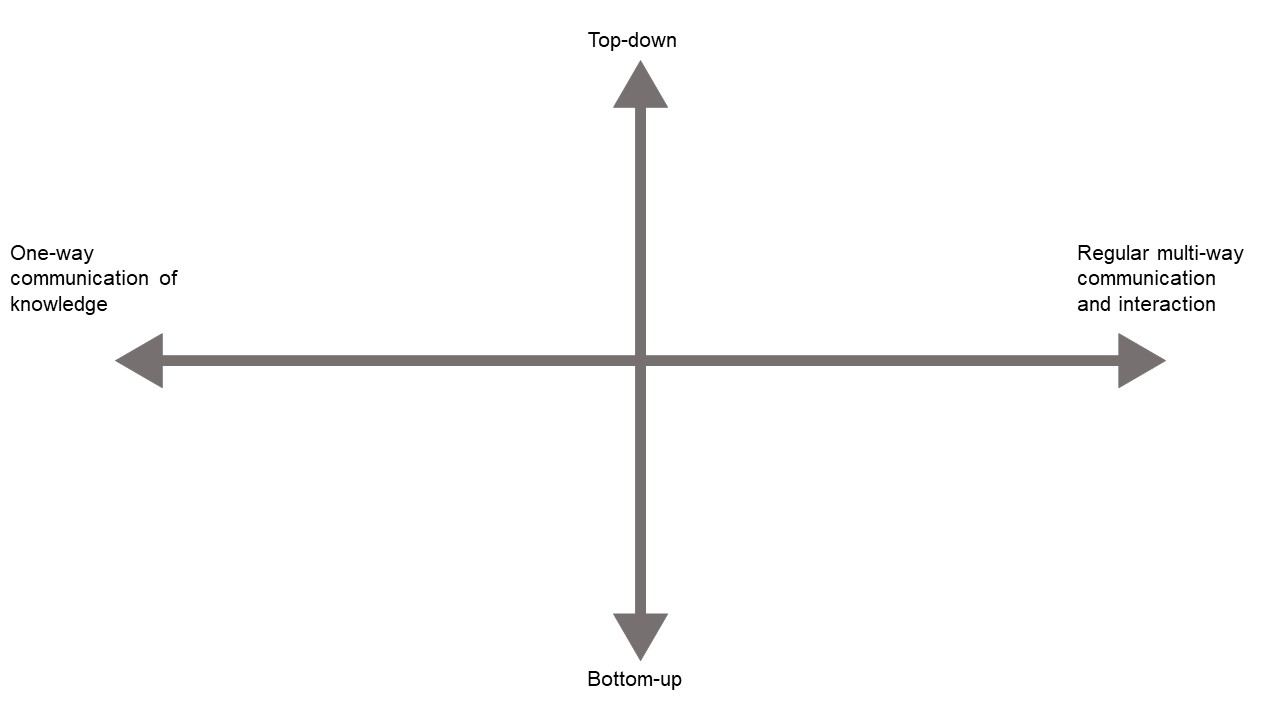


*Figure B1: Empty axis shown to interviewees to determine their implicit conceptualization of user engagement in their CIS, adapted from Vedeld et al. (2019).*

**9. Where would you say the CIS could be located within this graph? Why?**

*-Are you familiar with the concepts of co-production and co-creation of knowledge?*

*-Do you distinguish between these two concepts? How?*

If the interviewee is familiar with one or both of the concepts, the following questions will be asked:

*-Have (any of) the two concepts been considered in the development of the CIS?*

*-Did this happen explicitly? (Was e.g. co-creation defined as one of the main aims?)*

If the interviewee is NOT familiar with either of the concepts, the following questions will be asked:

*-How was user engagement discussed when developing the CIS design?*

*-What key terms were used? By whom?*

Appendix C: Code tree

Table C1: Code tree used to analyze the qualitative data collected for this research.

| *Implicit and explicit conceptualizations of user engagement by CIS providers* | *Characterization of user engagement during implementation CIS based on institutional design components* | *Other* |
| --- | --- | --- |
| -Positioning in matrix  -Definition user engagement CIS  -Definition co-production  -Definition co-creation  -Comments on co-production/co-creation  -Mentioning co-production/co-creation | -Design of arenas of interaction and collaboration  -Participation mechanisms  -Degree to which services decentralized and engage in on-site activities  *Includes*   - Traditional/local knowledge - Social learning at local level | -CIS activities  *Includes*   - Objectives of activities   -Challenges/limitations CIS  -Development of CIS |
